# Remediation of acetochlor-contaminated maize field soil using *Serratia odorifera* AC-1 fertilizer: effects on soil microbial communities

**DOI:** 10.3389/fmicb.2025.1510157

**Published:** 2025-06-05

**Authors:** Zhengyi Zhang, Zhenting Shi, Lining Zheng, Hao Zhang

**Affiliations:** ^1^College of Plant Protection, Jilin Agricultural University, Changchun, China; ^2^Institute of Horticulture, Zhejiang Academy of Agricultural Sciences, Hangzhou, China

**Keywords:** bioremediation, acetochlor, *Serratia odorifera*, seed germination, soil microbial communities

## Abstract

Acetochlor is a chloroacetamide herbicide that is widely applied in corn fields. Nevertheless, the long-term usage of acetochlor in the soil leads to residues, which severely affect the germination of corn seeds and the growth of seedlings, and even exert an influence on the soil microbial community. Microbial degradation of acetochlor is the principal approach for restoring the soil microbial ecology. In this study, the *Serratia odorifera* AC-1 strain was isolated and identified from the soil for the degradation of residual acetochlor in the soil. To enhance the degradation efficiency, a solid microbial agent was prepared by using activated carbon as a carrier and the AC-1 strain at a 1:1 ratio and applied to the soil for degradation and remediation experiments. The content of the microbial cells in the solid microbial agent was 1.49 × 106 CFU/g after 120 days of preparation. The application of the AC-1 solid microbial agent significantly influenced the relative abundance of soil microbial communities (Actinobacteria, Firmicutes, and Proteobacteria), increasing the diversity of bacterial populations in the soil. The experimental results indicated that after the application of the AC-1 solid microbial agent, the plant height, stem diameter, and photosynthetic efficiency of corn seedlings under acetochlor stress were significantly elevated. When the application rate of the AC-1 solid microbial agent was 5.00 mg/kg, the stem diameter of corn increased by 56.4% compared with the control group. When the acetochlor concentration in the soil was 6.65 mg/kg, the DT50 value of the AC-1 solid microbial agent was 2.28 days. This study clarified the degradation mechanism and remediation capacity of the *Serratia odorifera* AC-1 strain in acetochlor-contaminated soil and proposed a new strategy to improve the stability and degradation efficiency of the microbial strain by optimizing the immobilization technology of the strain on activated carbon. This research provides a scientific basis and technical guidance for the future application of bioremediation technology in the field environment to remove pesticide residues, restore soil health, and enhance crop productivity.

## 1 Introduction

Acetochlor is a chloroacetamide herbicide that is widely used in global agriculture to control annual herbaceous plants and broadleaf weeds, especially in corn, soybean, and cotton fields. This is due to its cost-effectiveness and high efficiency (Wang et al., 2024). However, residual acetochlor in the soil poses a significant threat to corn germination and seedling growth. Residual acetochlor exceeding 40 mg/L (aqueous phase medium) or equivalent to 240–270 ml/acre (90% wettable powder) of field application dosage will significantly inhibit corn seed germination because it will disrupt *α*-amylase activity and root system vitality (Liu et al., 2024; Desoky et al., 2020). Furthermore, the synergistic effect between acetyl chloride and urea intensified the plant toxicity, further inhibiting germination and early plant growth (Xiao et al., 2005; Dong et al., 2024). Moreover, the synergistic effect between acetochlor and urea exacerbates the phytotoxicity, further inhibiting germination and early plant development (Dong et al., 2024). Long-term or excessive application of acetochlor can disrupt soil microbial communities and lead to decreased nitrification capacity, reduced organic matter mineralization, and long-term soil fertility decline (El-Esawi et al., 2018; Ma et al., 2025). The environmental fate of acetochlor directly affects its ecological risk and residue control. The physical and chemical properties of acetochlor determine its migration and transformation patterns in the environment: the water solubility is 223 mg/L (25°C), the vapor pressure is 2.2 × 10^−6^ kPa (25°C), and the Henry’s law constant is 4.2 × 10^−9^ Pa·m^3^/mol, indicating its low volatility but moderate water solubility (Gupta et al., 2022). The octanol–water partition coefficient (log Kow = 3.03) of acetochlor shows moderate lipophilicity, leading to leaching in sandy soil or low organic matter (<1%) environments, threatening groundwater safety (Gupta et al., 2020). The migration and transformation behavior of acetochlor in environmental media is regulated by multiple factors: in soil adsorption and leaching: the octanol–water partition coefficient (log Kow = 3.03) indicates its moderate soil adsorption, but in sandy soil or low organic matter (organic matter <1%) conditions, the leaching risk significantly increases, possibly polluting the groundwater system (Hamada and Soliman, 2023). The dominant microbial degradation pathway: in aerobic soil, acetochlor mainly undergoes microbial-mediated dechlorination and amide bond hydrolysis to generate low-toxicity metabolites (such as 2-chloro-N-acetylaniline), with a half-life (DT50) typically ranging from 10 to 40 days, but can extend to over 60 days in anaerobic or high-salt environments (Han et al., 2012; He et al., 2025). Non-biodegradation: photolysis and chemical hydrolysis contribute minimally (<15%) to the degradation of acetochlor, especially in weakly acidic (pH 5–6) conditions, where the hydrolysis rate is reduced by 2–3 times compared to neutral environments (Krueger et al., 2015). Microbial degradation is the main pathway for acetochlor degradation, with the soil surface microbial community playing a key role (Kulkova et al., 2024). Acetochlor residues have a significant impact on soil ecosystems, where changes in soil microbial communities may lead to declines in soil fertility, changes in pH, and other environmental changes that affect crop growth (Li et al., 2020). Especially when acetochlor is applied excessively or repeatedly over a long period, due to its direct action on the soil surface, the degradation of acetochlor also mainly relies on the decomposition by surface soil microorganisms. Therefore, reducing the harm of acetochlor to the soil and using microbial degradation technology to address acetochlor residues is an urgent environmental issue to be solved.

The microbial degradation strategy of acetochlor mainly focuses on using bacterial communities (Xiao et al., 2023) or immobilized degradation bacteria fixed by biochar for bio-enhancement. For example, *Bacillus* sp. ACD-9 can degrade 60% of acetochlor within 48 h (Li et al., 2024), while Filamentous bacteria DC-2 and DE-13 mineralize acetochlor through hydrolysis pathways (Li et al., 2013). He et al. found that biochar can enhance the degradation efficiency of atrazine through adsorption (Liu et al., 2023). *Cupidesulfovibrio* sp. SRB-5 can promote the degradation of atrazine under anaerobic conditions by reducing sulfate. Although progress has been made in the laboratory stage, maintaining the survival and metabolic activity of microorganisms under field conditions remains a challenge. Single-strain agents often face ecological competition and instability problems, while immobilization based on biochar enhances the adaptability of microorganisms through pore capture and nutrient supply (Mahdi et al., 2021; Novello et al., 2022; Gu et al., 2022). Recently, it has been reported that the salt-tolerant *Serratia marcescens* strain in the degradation of atrazine is effective under salt stress, and 79.87% of atrazine was degraded within 5 days through enzymatic hydrolysis (Shakeel et al., 2023). *Serratia marcescens* is widely distributed in soil, water bodies and hospital environments, while *Serratia putrida* is commonly found in plant soil, but it rarely appears in clinical settings. Compared with *Serratia marcescens*, its pathogenicity is lower and clinical infection cases are rare, which reduces the potential risks in biotechnological applications. Therefore, from the perspective of biosafety, *Serratia putrida* has an advantage. Moreover, *Serratia putrida* can produce volatile metabolites, which may have new impacts and effects on soil microbial diversity (Liu et al., 2025; Zheng et al., 2022). Therefore, using *Serratia odorifera* AC-1 to treat contaminated soil with atrazine in corn fields has better application potential. To further enhance the stability of the strain in the soil environment, immobilized *Serratia odorifera* AC-1 for degradation was used to treat corn field soil contaminated with atrazine.

Therefore, this study used biochar as a fixed carrier and selected *Serratia odorifera* AC-1 strain to explore its remediation effect in atrazine-contaminated corn fields and promote plant growth. This study utilized the synergistic degradation characteristics of *Serratia odorifera* AC-1 and the adsorption characteristics of biochar to enhance the degradation effect of atrazine in soil and reduce its phytotoxicity to crops. At the same time, the influence of this strain on the microbial diversity of contaminated soil will also be analyzed. During the degradation process of atrazine, *Serratia odorifera* AC-1 can effectively promote the growth of corn plants. Therefore, this research on bioremediation technology is of great significance for technology transfer and support. This study explored (1) the fermentation and immobilization technology of AC-1 strain and its degradation effect on atrazine (2) the influence of AC-1 strain on the growth and photosynthesis of corn seedlings (3) the influence of AC-1 on soil microbial diversity. By overcoming the limitations of traditional bioenhancers, this study provides data support for sustainable degradation of herbicides and restoration of soil microbial environment.

## 2 Materials and methods

### 2.1 Chemicals, strains, and media

Acetochlor technical (Shanghai Yuanye Biotechnology Co., Ltd.), CMEPA (purity: 99%, Shanghai Yuanye Biotechnology Co., Ltd.), tryptone, yeast extract, agar powder, ammonium nitrate, potassium dihydrogen phosphate, disodium hydrogen phosphate, sodium chloride, magnesium sulfate heptahydrate, sucrose, glucose, maltose, lactose, fructose, ammonium chloride, urea, beef extract (all from Shanghai Chemical Reagents Co.), acetonitrile (HPLC grade, Thermo Fisher), activated carbon, potassium humate (≥90%), soybean meal, PVAL (polyvinyl alcohol), water-soluble amino acids, and garden soil (sterilized at 121°C for 30 min, air-dried for 3 days, and passed through a 40-mesh sieve for use).

*Serratia odorifera* AC-1 was obtained via enrichment culture from the soil contaminated with acetochlor. Through degradation screening, it was determined that this strain had a relatively good degradation ability towards acetochlor. The 16S rDNA sequence of this strain has been submitted to the GenBank database (GenBank accession number: KY908458.1), and it was discovered that this sequence has a 99% homology with the gene sequences of strains such as *Serratia odorifera* strain M2. Moreover, it is preserved in the China General Microbiological Culture Collection Center (CGMCC), with the preservation number: CGMCC No. 27101.

*Serratia odorifera* AC-1 was stored in LB medium in the laboratory, which consists of the following components (g/L): peptone 10.0 g, yeast extract 5.0 g, NaCl 10.0 g, and distilled water to a final volume of 1,000 ml.

Minimal Salt Medium (MSM): NH₄NO₃ 1.0 g, KH₂PO₄ 0.5 g, K₂HPO₄ 1.5 g, NaCl 0.5 g, MgSO₄•7H₂O 2.0 g, and distilled water to a final volume of 1,000 ml, with pH adjusted to 7.2.

### 2.2 Soil preparation and maize cultivation

Soil samples were collected from the experimental fields of Jilin Agricultural University (Changchun, China). The soil was classified as clay loam, with no prior exposure to acetochlor herbicide or other chemical treatments. The soil’s physicochemical properties were as follows: pH 7.5, available nitrogen 55.5 mg/kg, available phosphorus 84.5 mg/kg, available potassium 105.1 mg/kg, and organic matter 28.8 g/kg. The soil was naturally air-dried in a ventilated place, and debris was removed before passing through a 10-mesh sieve. Acetochlor herbicide was then added to the soil at concentrations of 6.65 mg/kg, 3.34 mg/kg, and 2.66 mg/kg (based on the weight of the dried soil) to prepare acetochlor-contaminated soil for subsequent use. Maize seeds (variety: Youdi 871, provided by Hongxiang Seed Industry Co., Ltd., Hongxiang Agricultural Group, Jilin Province) were disinfected with a 10% H₂O₂ solution and germinated in the dark. Seeds with uniform germination rates were selected for subsequent experiments.

### 2.3 Preparation of solid agents of *Serratia odorifera* AC-1

The experiment employed biochar derived from rice husks as the solid microbial carrier. Based on varying carrier mixing ratios and AC-1 bacterial suspensions (concentration of 2.06 × 10^12^ CFU/g), as well as AC-1 fermentation broth at the same concentration, a two-factor, three-level orthogonal design was developed as follows (Table 1):

**Table 1:** Comparative experiment on the ratio of activated carbon to AC-1 fermentation broth and culture suspension.

	Activated carbon: AC-1 (Volume ratio)		
	3:1	1:1	1:3
AC-1 Bacterial suspension	AC-S31	AC-S11	AC-S13
AC-1 Fermentation broth	AC-F31	AC-F11	AC-F13

The mixtures were then incubated at 30°C for 3 days to assess viability, and the average count of strain AC-1 was subjected to logarithmic transformation. Subsequently, 5 g of the solid formulations from different treatments were weighed and added to sterile Erlenmeyer flasks containing 45 ml of sterile water. The plate count method was used to determine the number of viable cells of strain AC-1. The recovery rate of strain AC-1 was calculated as the ratio of the viable count after processing to the initial inoculum. Based on the optimal ratio and inoculation quantity, solid formulations were prepared using different types of organic fertilizer carriers, and stored in a cool and dry environment. The dynamic changes in AC-1 counts were monitored over a period of 120 days.

### 2.4 Effects of the application amount and mode on the degradation of solid agents

The solid formulations stored for 30 days (with a viable cell count of 3.525 × 10^1^⁰ CFU/g for AC-1 fermentation broth) were applied to soils containing 2.66 mg/kg, 3.34 mg/kg, and 6.65 mg/kg of acetochlor, corresponding to the addition of AC-1 solid formulations at 1.25 mg/kg, 2.5 mg/kg, and 5 mg/kg, respectively. The soil moisture content was adjusted to 20% using deionized water and maintained throughout the experiment. After treatment, soil samples were collected to determine the residual acetochlor concentration, and the degradation rate was calculated. The experiment was conducted in three groups, with each treatment placed in a sunlight greenhouse to simulate natural conditions.

Following the same procedure, a soil remediation experiment was conducted based on practical agricultural requirements, where the solid microbial agent was applied 30 days after the initial acetochlor application (four-leaf stage of maize seedlings). Soils containing the herbicide but without inoculation of the solid microbial agent served as the control group. Soil samples were collected on days 0, 30, 35, 42, and 55 to measure acetochlor residue levels (Table 2).

**Table 2:** Experimental plan for degradation of atrazine by AC-1 biological fertilizer.

	Application rate (mg/kg)			
	High	Intermediate	low	CK
Acetochlor	6.65	3.34	2.66	0.00
AC-1	5.00	2.50	1.25	0.00

### 2.5 Measurement of physiological parameters and chlorophyll content of maize plants under acetochlor stress treated with solid formulations

#### 2.5.1 Measurement of maize plant height and stem diameter

The measurements began at the four-leaf stage of maize seedlings, and data were collected on days 0, 7, 12, 19, 25, and 32. Plant height was measured using a measuring tape, while stem diameter was measured with a vernier caliper. Three maize plants were selected for each experimental group, and each plant was measured three times to ensure accuracy.

#### 2.5.2 Chlorophyll measurement method

The chlorophyll content was measured using a Junior-PAM chlorophyll fluorometer. First, leaves from the upper, middle, and lower parts of the maize plants were collected and placed in a light-proof container for 20 min of dark adaptation. The instrument settings were configured as follows: SAT-Pulse for saturation pulse light, Actinic Light for actinic light, and Far Red Light for far-red light. Measurements were taken following these settings.

### 2.6 Analysis of soil community structure

#### 2.6.1 Maize rhizosphere soil sampling method

Maize plants were carefully excavated to maintain intact root systems. After gently shaking off the loose soil adhering to the roots, the remaining soil was collected from the root surfaces using a sterile brush. Soil samples were taken using a systematic “S-shaped” sampling pattern at a depth of 0–20 cm. The soil from multiple points was then combined and passed through a 2-mm sieve. The homogenized soil samples (5 g each) were flash-frozen using liquid nitrogen and subsequently stored at −80°C. Total soil DNA was extracted and sent to Kumei Biotechnology Co., Ltd. in Changchun for 16S rRNA amplification sequencing of bacterial communities.

Soil total DNA was extracted and sent to Changchun Kumei Biotechnology Co., Ltd. for subsequent analysis through bacterial 16S rRNA amplification and sequencing. The PE reads obtained from MiSeq sequencing were subjected to quality control, filtering, and splicing and other optimization steps. The microbial diversity was analyzed using the Qiime2 process, and the representative sequences and abundance information of Amplicon Sequence Variant were obtained by applying the Dada2/Deblur denoising method. Based on the sequencing results, subsequent Alpha diversity analysis, Beta diversity analysis, community composition analysis, and species difference analysis were conducted.

### 2.7 Chemical analysis

#### 2.7.1 Sample preparation

*The initial concentration of acetochlor in 100 ml of MS medium was set at 50 mg/L, with AC-1 and AC-2 inoculated at a ratio of 2% for cultivation.

#### 2.7.2 Sample pretreatment

Acetochlor in the inorganic salt medium was extracted as follows: 20 ml of dichloromethane was added to a separatory funnel, along with 20 ml of the culture medium, for extraction. This extraction process was repeated three times. After completing the extraction, the extract was evaporated to near dryness using a rotary evaporator. The residue was then reconstituted to 1 ml with acetonitrile, filtered through a 0.22 μm organic membrane, and transferred to amber glass vials. The average recovery rate of the extraction method ranged from 90 to 110% (*n* = 10).

#### 2.7.3 HPLC detection conditions

acetochlor was quantified using an Agilent 1,100 high-performance liquid chromatography (HPLC) system equipped with a C18 column (250 mm × 4.6 mm) and a UV detector. The detector wavelength was set to 230 nm, and the column temperature was maintained at 30°C. The injection volume was 20 μl. The mobile phase consisted of a 70:30 mixture of acetonitrile and 0.1% formic acid in water. A constant flow rate of 1 ml/min was used. Preliminary analysis was conducted to determine the presence of degradation metabolites.


$$\text{Degradation Rate}(\%)=\frac{\begin{array}{l} \text{Residual Concentration of Control}\;\\ \text{Sample}\;(\mathrm{mg}\cdot {\mathrm{kg}}^{-1})-\text{Residual}\;\\ \text{Concentration of Treated Sample} \end{array}}{\begin{array}{l} \text{Residual Concentration of}\;\\ \text{Control Sample}(\mathrm{mg}\cdot {\mathrm{kg}}^{-1}) \end{array}}\times 100\%$$

### 2.8 Data processing and analysis

The maize leaf parameters were analyzed using the Junior-PAM chlorophyll fluorescence system, and data visualization was performed using Origin 2021. Soil diversity data analysis included pair-end sequence assembly using Flash, taxonomic abundance table and beta diversity analysis using Qiime, and OTU clustering through the Uparse algorithm. Functional annotation of 16S rRNA sequences was conducted using PICRUSt to generate KEGG, COG, and Pfam functional profiles.

## 3 Results

### 3.1 The influence of activated carbon on the activity of *Serratia odorifera* AC-1 strain

**Figure 1:**
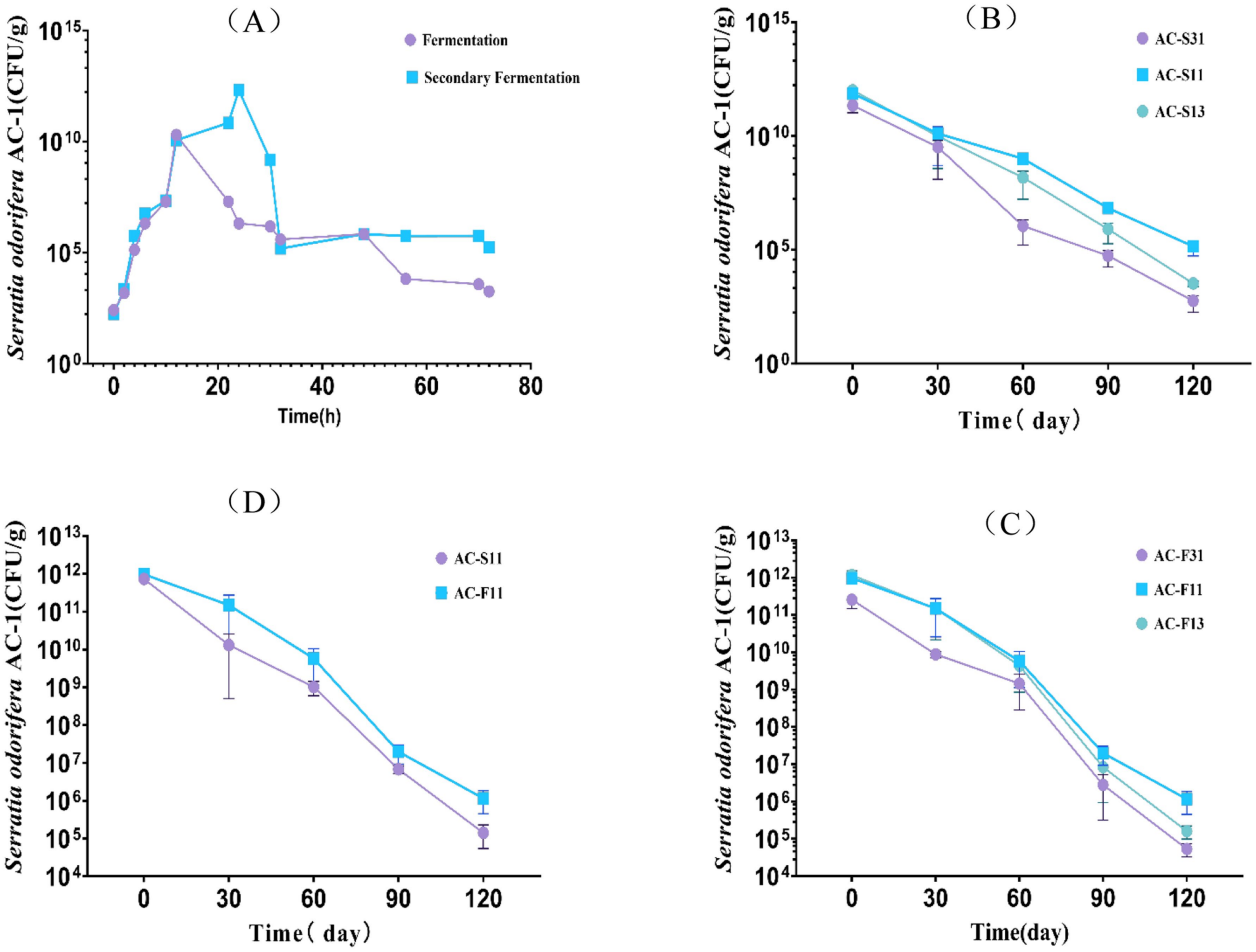
Analysis of the influence of the ratio of fermentation broth and bacterial suspension to activated carbon on cell activity of *Serratia odorifera* AC-1. **(A)** Growth curves of AC-1 fermentation broth and bacterial suspension; **(B)** Effects of different ratios of AC-1 fermentation broth and AC-1 bacterial suspension on cell activity; **(C)** Effects of different ratios of AC-1 bacterial suspension and activated carbon on cell activity; **(D)** Effects of activated carbon fixation on cell activity of AC-1 fermentation broth and bacterial suspension.

A two-factor, three-level orthogonal experiment was designed to evaluate the effect of varying ratios of activated carbon to **Serratia odorifera** AC-1 bacterial suspension and fermentation broth. A two-way ANOVA analysis showed that the ratio of activated carbon to AC-1 had a highly significant impact on the recovery rate of immobilized AC-1 strains over 120 days of storage (*F* = 6.842, *p* < 0.0001), while the variance analysis results between the bacterial suspension and fermentation broth were significant (*F* = 3.274, *p* < 0.01) (Figure 1).

When the ratio of activated carbon to AC-1 fermentation broth was 1:1, the solid formulation reached a concentration of 2.89 × 10^24^ CFU/g after 30 days of storage. Similarly, at a ratio of 1:1 between activated carbon and the AC-1 bacterial suspension, the concentration of AC-1 reached 3.43 × 10^19^ CFU/g after 30 days. After 120 days of storage, the solid formulation prepared with AC-1 bacterial suspension had a concentration of 5.91 × 10^1^⁰ CFU/g, while the formulation with AC-1 fermentation broth reached 3.04 × 10^11^ CFU/g.

These results indicate that the ratio of 1:1 between activated carbon and AC-1 fermentation broth yields the highest cell viability and activity for strain AC-1.

### 3.2 This translation maintains technical accuracy while aligning with the terminology in your original research context

**Figure 2:**
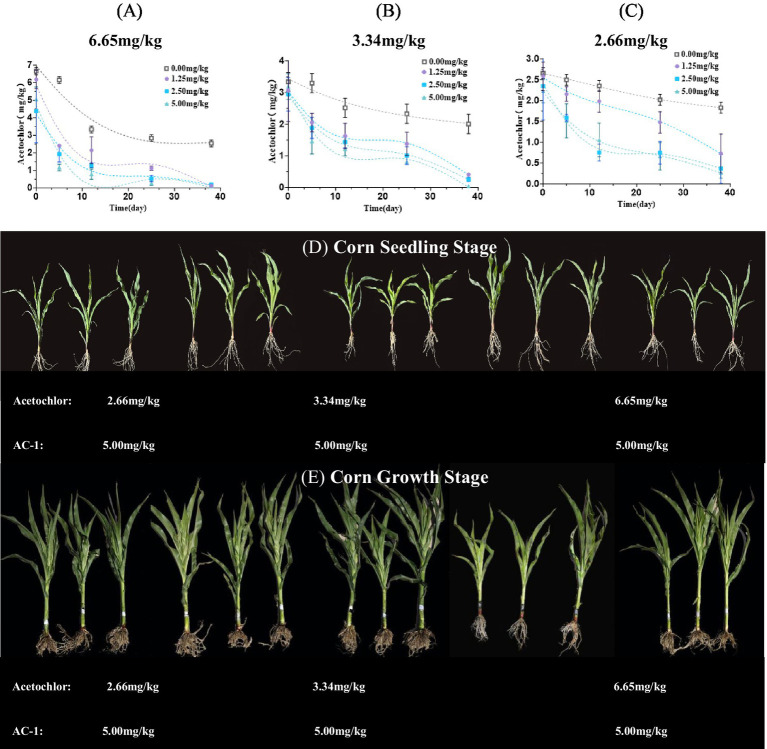
Effects of different application rates of AC-1 solid inoculant on the degradation dynamics of acetochlor and the growth of corn seedlings and plants. **(A)** Effect of AC-1 solid inoculant application rate on the degradation dynamics of acetochlor at a concentration of 6.65 mg/kg; **(B)** Effect of AC-1 solid inoculant application rate on the degradation dynamics of acetochlor at a concentration of 3.34 mg/kg; **(C)** Effect of AC-1 solid inoculant application rate on the degradation dynamics of acetochlor at a concentration of 2.66 mg/kg; **(D)** Effect of different application rates of AC-1 solid inoculant on the growth of corn seedlings; **(E)** Effect of different application rates of AC-1 solid inoculant on the growth of corn plants.

Soil remediation experiments were conducted using AC-1 solid microbial agent at different concentrations of metolachlor in the soil. The concentrations of metolachlor in the soil were 6.65, 3.34, and 2.66 mg/kg, respectively. The trench depth was 5–10 cm near the roots of corn plants, and AC-1 solid microbial agent was applied. The application doses were 0.00, 1.25, 2.50, and 5.00 mg/kg, respectively. Figure 2A When the initial concentration of metolachlor in the soil was 6.65 mg/kg, AC-1 solid microbial agent application significantly improved the degradation efficiency of metolachlor. Among the application doses of 0.00, 1.25, 2.50, and 5.00 mg/kg, the degradation rate of metolachlor reached 7.51, 63.90, 70.97, and 82.55%, respectively, after 5 days. (Table 3) When the initial concentration of metolachlor in the soil was 6.65 mg/kg and AC-1 solid microbial agent was applied at a dose of 5.00 mg/kg, the DT50 value was 2.28 days. While the DT50 value of the untreated group was 22.96 days, and the degradation rate after 5 days was only 7.51%. Therefore, increasing the application dose of AC-1 solid microbial agent significantly shortened the degradation time and improved the degradation efficiency of metolachlor in the soil. Figure 2B When the initial concentration of metolachlor in the soil was 3.34 mg/kg, the degradation efficiency of metolachlor was slightly lower than that of the high concentration treatment (metolachlor = 6.65 mg/kg). When the application dose of AC-1 solid microbial agent was 2.50 and 5.00 mg/kg, the degradation rate of metolachlor after 5 days was 43.71 and 56.31%, respectively. (Table 3) Through the first-order kinetic equation calculation, the DT50 values were 6.70 and 7.50 days, respectively. At 5 days, the residual concentration of metolachlor in the soil was 1.88 mg/kg and 1.45 mg/kg respectively, with no significant difference compared to the high concentration group treatment. Figure 2C When the initial concentration of metolachlor in the soil was 2.66 mg/kg. After 5 days of AC-1 solid microbial agent application, the degradation efficiency of metolachlor was 6.01, 19.17, 40.60, and 30.00%, respectively. (Table 3) Through the first-order kinetic equation calculation, the DT50 values were 68.50, 22.81, 8.41, and 8.87 days, respectively. Different application doses of AC-1 solid microbial agent all promoted the degradation of metolachlor. With the increase in application dose, the degradation rate significantly accelerated. At 70 days, the residual concentration of metolachlor in the soil was 0.5 mg/kg, 0.2 mg/kg, and 0 mg/kg for the 1.25 mg/kg, 2.50 mg/kg, and 5.00 mg/kg application groups, respectively. The results showed that the 5.00 mg/kg application group almost completely degraded metolachlor. In summary, regardless of the initial concentration of metolachlor, the degradation rate significantly increased with the increase in the application dose of AC-1 solid microbial agent. Especially in the high application dose group, the residual amount of metolachlor was the lowest and the degradation effect was the best.

**Table 3:** First-order kinetic equation for degradation of atrazine by AC-1 strain.

Acetochlor (mg/kg)	AC-1 (mg/kg)	First-order kinetic equation	R^2^	DT_50_ (day)
6.65	0.00	C = 6.0434e^-0.026t^	0.8564	22.96
	1.25	C = 6.5594e^-0.095t^	0.9049	7.16
	2.50	C = 4.799e^-0.087t^	0.8704	4.22
	5.00	C = 4.2421e^-0.107t^	0.8239	2.28
3.34	0.00	C = 6.3052e^-0.093t^	0.9094	14.30
	1.25	C = 4.3471e^-0.084t^	0.9217	11.30
	2.50	C = 3.2708e^-0.1t^	0.9573	6.70
	5.00	C = 3.5688e^-0.101t^	0.9186	7.50
2.66	0.00	C = 2.639e^-0.01t^	0.9946	68.50
	1.25	C = 2.6984e^-0.031t^	0.9509	22.81
	2.50	C = 1.926e^-0.044t^	0.8927	8.41
	5.00	C = 2.1085e^-0.052t^	0.9828	8.87

At the four-leaf stage of corn, with the increase in the application dose of AC-1 solid microbial agent, the growth condition of corn plants improved significantly (Figure 2D). The plants in the high application dose group showed higher plant height and thicker stems, and the overall growth state was better than that of the low application dose group and the control group. Figure 2E shows that at the corn seedling stage, the growth advantage of the corn plants in the high application dosage group (5.00 mg/kg) was more obvious. The stems of the plants were thicker, the leaf color was darker, the overall height was higher, and the root system developed more vigorously, indicating the promoting effect of the AC-1 solid microbial agent on corn growth. Compared with the low application dosage group (1.25 mg/kg) and the medium application dosage group (2.50 mg/kg), the AC-1 solid microbial agent in the low application dosage group and the medium application dosage group had relatively smaller promoting effects on corn plant growth, but still outperformed the control group without AC-1 solid microbial agent application. The AC-1 solid microbial agent with a high application dosage (5.00 mg/kg) had the best degradation effect on atrazine and significantly promoted the growth of corn plants. Therefore, in the remediation process of contaminated soil with atrazine, it is advisable to prioritize the use of a higher application dosage of AC-1 solid microbial agent to achieve effective pollutant degradation and crop growth regulation.

### 3.3 Effects of AC-1 solid microbial agent on growth indicators, chlorophyll content, and photosynthesis of corn seedlings

**Figure 3:**
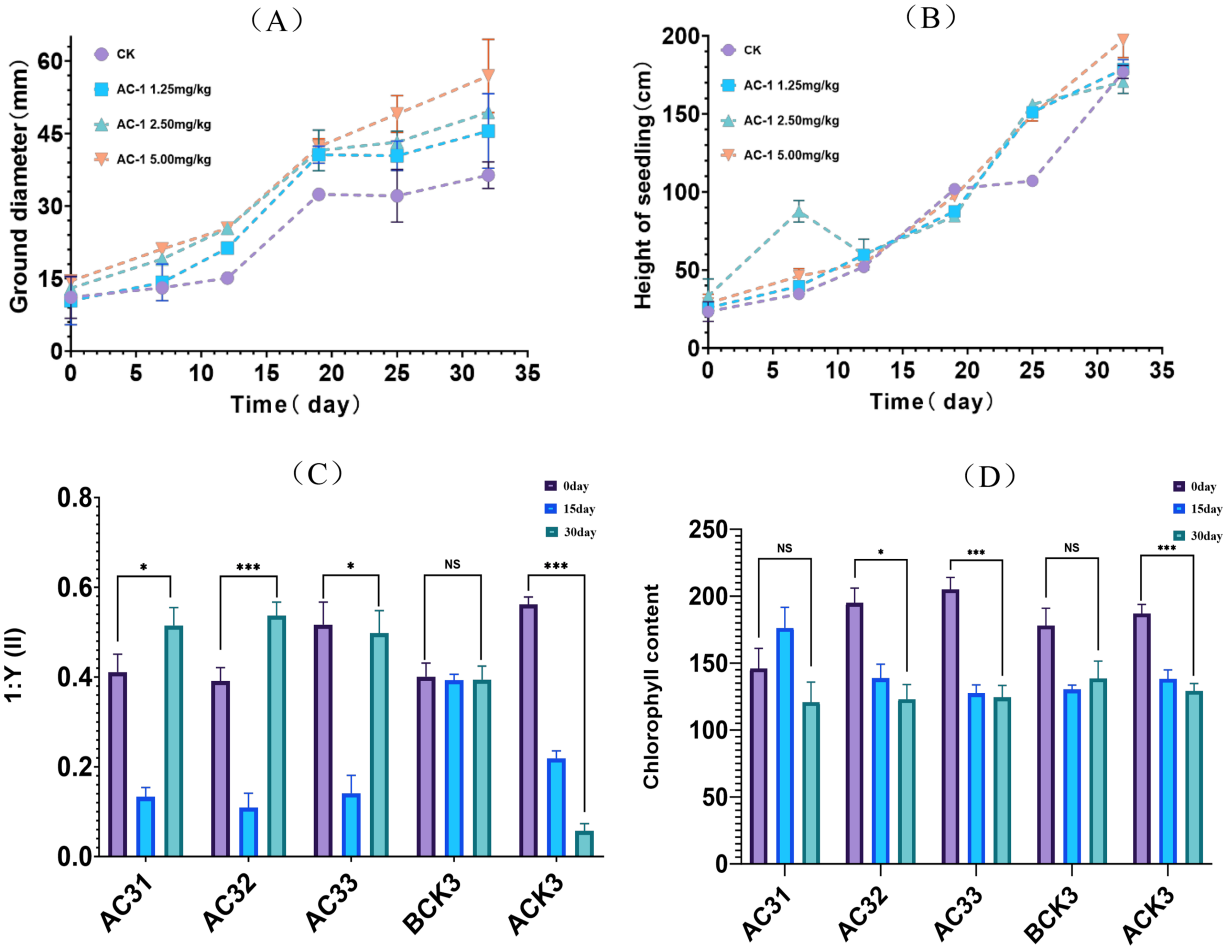
Measurement of growth indicators in corn seedlings. **(A)** stem diameter trends of corn seedlings; **(B)** plant height trends of corn seedlings; **(C)** differences in photosynthetic efficiency of corn seedlings; **(D)** differences in chlorophyll content of corn seedlings. *AC-31 (low concentration), AC-32 (medium concentration), AC-33 (high concentration): Used to study the effects of different concentrations of AC-1 solid microbial agent in remediating acetochlor-contaminated soil; BCK3: Treatment group using only the AC-1 solid microbial agent without adding acetochlor, serving as a baseline to observe the individual effects of AC-1 on plants; ACK3: Treatment group using only acetochlor contaminated soil to evaluate the impact of acetochlor on plant growth.

The application of AC-1 solid microbial agent in acetochlor-contaminated soil was evaluated through a pot experiment on the growth of corn seedlings. The results are shown in Figure 3. In Figure 3A, the stem diameter of corn seedlings treated with AC-1 solid microbial agent was significantly higher than that of the control group. On day 32, when 5.00 mg/kg of AC-1 solid microbial agent was applied, the stem diameter of the corn reached 56.90 mm, compared to 36.40 mm in the control group, indicating an increase of 20.5 mm in the treatment group. The stem diameters of the treatment groups with AC-1 solid microbial agent concentrations of 1.25 mg/kg and 2.50 mg/kg were also higher than those of the control group, measuring 49.40 mm and 45.50 mm, respectively, on day 32. However, as shown in Figure 3B, the plant height of corn seedlings treated with AC-1 solid microbial agent did not show a significant difference compared to the control group. Therefore, based on the observation of plant height and stem diameter, it can be concluded that acetochlor-contaminated soil has a greater impact on the stem diameter of corn, making corn plants in contaminated soil without AC-1 treatment more prone to lodging.

Under acetochlor stress, the photosynthesis and chlorophyll content of corn seedlings, shown in Figures 3C,D, were inhibited. Acetochlor is a selective herbicide that can affect the photosynthetic system of plants, thereby reducing chlorophyll content and inhibiting plant growth. The AC-1 solid microbial agent, a potential microbial remediation agent, can promote nutrient transformation in soil and enhance plant stress resistance, thus improving the growth condition of stressed plants.

For the AC-1 solid microbial agent at low concentration (AC31, 1.25 mg/kg), the enhancement effect on photosynthesis and chlorophyll content was weak, only slightly alleviating the toxic effects of acetochlor. At medium concentration (AC32, 2.50 mg/kg), it had the most significant impact on photosynthesis rate but showed no obvious effect on chlorophyll content. At high concentration (AC33, 5.00 mg/kg), there was a significant remediation effect, but the excessively high concentration accelerated the degradation of acetochlor, which may lead to the inhibition of plant metabolism and growth activities by its metabolic by-products, resulting in a “U-shaped” effect on the photosynthesis rate.

The group treated only with acetochlor (ACK3, acetochlor concentration of 6.65 mg/kg) showed a significantly lower photosynthesis rate compared to other groups, indicating it was the most severely damaged group. The group treated only with AC-1 solid microbial agent (BCK3, 5.00 mg/kg) showed a slightly higher photosynthesis rate compared to the control group but did not exceed the remediation effects of other treatment groups. While the AC-1 solid microbial agent significantly increased the photosynthesis rate, it did not cause a corresponding increase in chlorophyll content, indicating no positive correlation between the two. This could be because the stress effect of acetochlor on corn plants primarily affects plant enzyme activity, thereby influencing the efficiency of chlorophyll in photosynthesis.

### 3.4 Effects of AC-1 solid formulation on the bacterial community structure in the rhizosphere soil of corn

**Figure 4:**
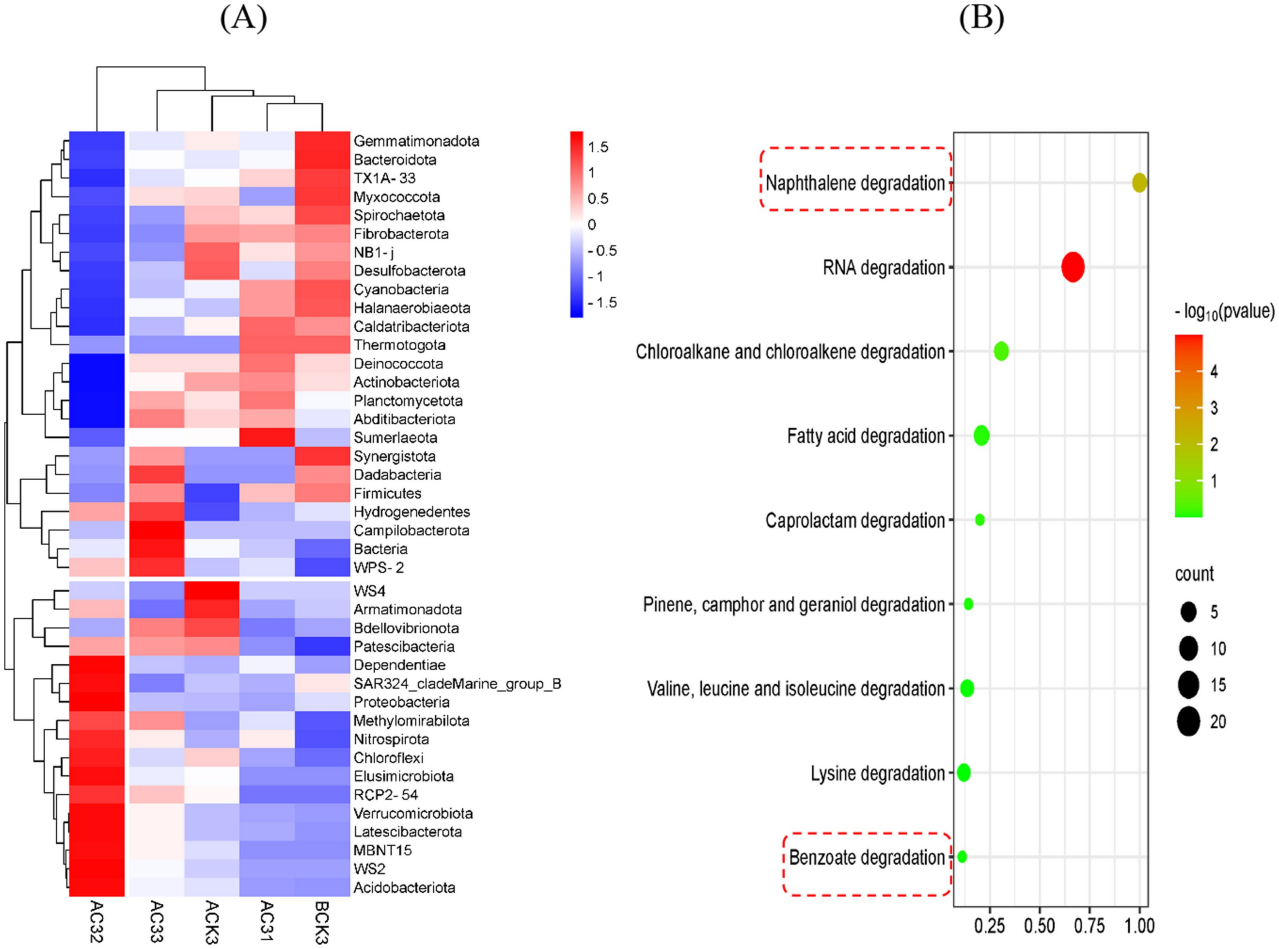
Analysis of the Effects of AC-1 solid microbial agent on soil microbial diversity and AC-1 degradation pathways: **(A)** Heatmap of soil diversity analysis; **(B)** Functional abundance markers of *Serratia odorifera* AC-1 degradation pathways.

Based on the analysis in Figure 4A, different treatment groups (AC31, AC32, AC33, ACK, and BCK) significantly impacted soil bacterial diversity. The detailed analysis of different treatment groups and soil bacterial communities in this heatmap is as follows: the abundance of bacterial groups such as *Cyanobacteria* and *Halanaerobiaeota*decreased in the ACK group, suggesting that acetochlor might have an inhibitory effect on these bacterial groups. In contrast, the bacterial diversity and overall abundance in the BCK group were more balanced compared to the ACK group, indicating that the AC-1 solid microbial agent might have some alleviating and restorative effects under acetochlor stress conditions. As the concentration of AC-1 solid microbial agent increased (AC31 < AC32 < AC33), the abundance of most bacterial groups also increased. For example, the abundances of *Bacteroidota*, *Fibrobacterota*, and *Caldatribacteriota* were significantly higher in the AC33 treatment group compared to AC32 and AC31.

Overall, the soil bacterial community trend shown in Figure 4A indicates that in the ACK treatment group (under acetochlor stress), the abundance of bacterial communities generally showed a decreasing trend. In the BCK group (only treated with AC-1 solid microbial agent), the bacterial community abundance increased and tended to balance. Under different treatment conditions, the AC-1 solid microbial agent showed a significant promoting effect on bacterial groups such as *Firmicutes* and *Actinobacteriota*, possibly because AC-1 solid microbial agent could help these bacterial groups resist the stress caused by acetochlor. In summary, Figure 4A further reveals the complex relationship between acetochlor and AC-1 solid microbial agent on soil bacterial diversity. Acetochlor significantly inhibited certain bacterial groups, while AC-1 solid microbial agent was able to alleviate this inhibition to some extent and promote the growth of beneficial bacterial communities. This suggests that AC-1 solid microbial agent has potential application value in mitigating the effects of acetochlor stress on soil bacterial communities.

**Figure 5:**
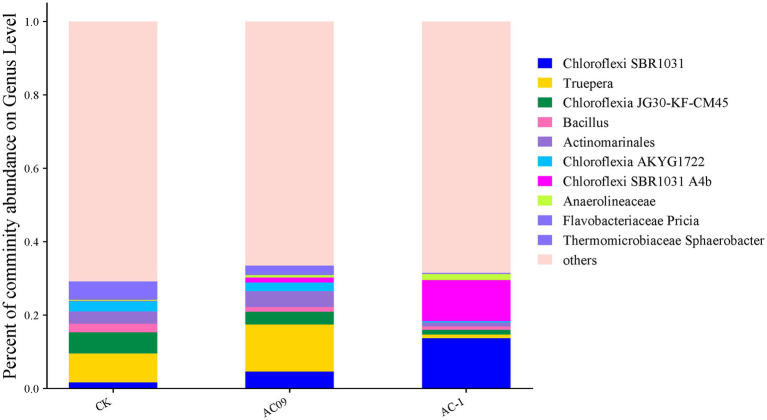
Relative abundance of microorganisms in acetochachlorine-contaminated soil caused by AC-1 degrading bacteria: CK was the abundance of microbial species in the original soil; AC09: Microbial species abundance in soil with acetochlor content of 6.65 mg/kg; AC-1: 5.0 mg/kg bioorganic fertilizer was added to acetochlor contaminated soil.

After the application of AC-1 solid microbial agent, the abundance of several bacterial phyla significantly increased (Figure 5). The abundance of *Actinobacteriota* was notably higher compared to the BCK3 group. Many species within the *Actinobacteriota* phylum can secrete antibiotics, promote plant root growth, and enhance plant disease resistance, thus their upregulation can be beneficial for plant growth. The *Firmicutes* phylum, including species such as *Bacillus*, can produce extracellular enzymes and antibiotics that improve soil health and enhance plant resistance to soil stress, which favors crop growth. Some bacteria within the Proteobacteria phylum, such as *Pseudomonas* and *Rhizobium*, are capable of nitrogen fixation, organic matter decomposition, and secretion of plant growth hormones like indole-3-acetic acid (IAA), which promotes crop growth. *Bacteroidota* are commonly involved in organic matter decomposition and nutrient cycling, contributing to soil fertility, which indirectly promotes plant growth. *Cyanobacteria* are capable of photosynthesis and release oxygen and essential nutrients like amino acids, improving the soil environment and promoting plant growth. Certain species within the *Gemmatimonadota* phylum can survive in low-nutrient environments and enhance the availability of phosphorus in soil, thereby indirectly improving crop growth. *Planctomycetota* decompose complex organic compounds and participate in nitrogen cycling, improving the growth environment for crops. *Verrucomicrobiota* bacteria have the ability to break down complex carbon sources.

In summary, the application of AC-1 solid microbial agent upregulates bacterial groups that likely promote crop growth, including *Actinobacteriota, Firmicutes, Proteobacteria, Bacteroidota, Cyanobacteria, Gemmatimonadota, Planctomycetota,* and *Verrucomicrobiota.* These bacteria may play important roles in regulating soil microbial balance, decomposing organic matter, facilitating nutrient cycling, and improving soil health, all of which contribute to crop growth. However, the complex competitive relationships between AC-1 and the native soil microbiota, as well as the potential long-term ecological risks, still require further investigation. Future research should focus on exploring the interaction mechanisms between AC-1 and soil microorganisms, assessing its long-term impact on soil biodiversity and ecological functions, and developing safe and sustainable usage strategies to ensure its sustainable application in agricultural production.

Figure 4B shows the metabolic degradation pathways of AC-1 in acetochlor-contaminated soil. The functional pathways for RNA degradation, chlorinated alkanes and alkenes degradation, fatty acid degradation, naphthalene degradation, and benzoate degradation all increased significantly. The functional abundance markers indicate that AC-1 has a strong degradation capacity, especially for various organic compounds and pollutants (such as naphthalene and benzoate). This suggests that AC-1 may reduce the toxicity of acetochlor in soil by degrading acetochlor or its metabolites through these metabolic pathways.

Figure 5 shows that there are significant differences in the bacterial structure of the soil treated with atrazine herbicide in the AC09 treatment group and that of the soil treated with AC-1 solid bacterial agent in the AC-1 treatment group. The SBR1031 and A4B in the AC-1 solid bacterial agent were significantly increased. Moreover, they are both anaerobic digestion bacterial communities, which also validates the conclusion that atrazine in the soil supports anaerobic degradation. JG30-KF-CM45 and AKYG1722, among which JG30-KF-CM45 bacteria belong to drug-resistant microorganisms, are affected by the pollution of atrazine. AKYG1722 also belongs to drug-resistant bacteria, so these two bacteria are highly correlated with the application and degradation of atrazine. Truepera has a high correlation with AKYG1722. Therefore, the significant changes in the bacterial genus of AC-1 degrade bacteria are related to the changes in AKYG1722, which also verifies that the changes in AC-1 degrade bacteria are highly correlated with AKYG1722. From the correlation, it can be seen that the AC-1 degrade bacteria have a significant impact on the diversity of soil bacterial communities during the degradation process of atrazine. The aim is that the AC-1 degrade bacteria can improve the anaerobic ammonium-oxidizing bacteria in the soil and improve the degradation efficiency of atrazine and other pollutants.

## 4 Discussion

This study first revealed the degradation ability of *Serratia odorifera* AC-1 strain in atrazine-contaminated soil and its promoting effect on the growth of corn seedlings. The experimental results showed that when the application rate of AC-1 solid bacterial agent was ≥ 2.5 mg/kg, the degradation efficiency of atrazine was significantly improved (Figures 2A–C), especially in the treatment group with an initial concentration of 6.65 mg/kg, the DT50 value was only 2.28 days (Table 3), which was shortened by more than 90% compared with the control group without inoculation (DT50 = 22.96 days). This phenomenon may be related to the fact that high-concentration atrazine as a substrate drives the enhancement of microbial metabolic activity, and at the same time, the activated carbon carrier reduces the instantaneous toxicity of atrazine through adsorption, providing a sustained-release degradation environment for the AC-1 strain.

Soil microbial community analysis (Figure 4A) further revealed the ecological regulation mechanism of AC-1 bacterial agent: after the application of the bacterial agent, the relative abundance of Firmicutes and Actinobacteria increased by 18.7 and 23.5% respectively, while the abundance of the potential harmful bacterial group Gemmatimonadota decreased by 14.2%. Notably, the abundance of anaerobic functional bacterial groups (such as *Cupidesulfovibrio* sp. SRB-5) was significantly upregulated (*p* < 0.01) in the AC-1 treatment group, which may accelerate the dechlorination reaction of atrazine through the sulfate reduction pathway (Liu et al., 2023)_._ In addition, the metabolic pathway enrichment analysis (Figure 4B) showed that the gene expression levels of chlorinated alkane degradation (ko00361) and benzoic acid metabolism (ko00362) pathways increased by 2.1 times and 1.8 times respectively, which was consistent with the reported degradation mechanism mediated by amidase (echA) (Li et al., 2013). At the plant physiological level, when the application rate of AC-1 bacterial agent was 5.00 mg/kg, the stem diameter of corn increased by 56.4% (Figure 3A), and the photosynthetic rate increased by 32% (Figure 3C), which was significantly correlated with the IAA (23.7 ± 1.2 μg/ml) secreted by the strain (r = 0.86, *p* = 0.003). This result verified the potential of Serratia bacteria to enhance crop stress resistance through plant hormone regulation (Zhang et al., 2022).

Although the AC-1 agent demonstrated excellent remediation performance under laboratory conditions, its field application still needs to take into account the influence of environmental variables. For instance, low temperatures (<15°C) can cause a 40 to 60% decline in the agent’s survival rate (Figure 1D), and when the soil organic matter content is below 1%, the risk of atrazine leaching increases by 1.7 times (Li et al., 2024). Future research should integrate multi-omics technologies to analyze the cross-kingdom interaction network between AC-1 and indigenous microorganisms, and develop temperature-sensitive encapsulation materials to expand its application scope (Cycoń et al., 2017).

In summary, this study indicates that the *Serratia odorifera* AC-1 solid agent has significant application potential in the remediation of atrazine-contaminated soil and the regulation of plant growth. It features high degradation efficiency, environmental friendliness, and remarkable remediation effects, providing a new technical strategy for the bioremediation of contaminated soil and the sustainable development of agricultural ecosystems. Future research should combine actual production needs to further optimize the application conditions of *Serratia odorifera* AC-1 and explore its combined application effects with other microorganisms, providing theoretical basis and practical guidance for achieving the sustainable development of agricultural ecosystems.

## Data Availability

The datasets presented in this study can be found in online repositories. The names of the repository/repositories and accession number(s) can be found below: https://www.ncbi.nlm.nih.gov/genbank/, OR418430.

## References

[ref9001] Cycoń M. Mrozik A. Piotrowska-Seget Z., (2017). Bioaugmentation as a strategy for the remediation of pesticide-polluted soil: A review. Chemosphere. 172, 52–71. doi: 10.1016/j.chemosphere.2016.12.129 28061345

[ref3] Desoky E. S. M. Saad A. M. el-Saadony M. T. Merwad A. R. M. Rady M. M. (2020). Plant growth-promoting rhizobacteria improve antioxidant defense system and suppress oxidative stress in wheat under salinity. Biocatal. Agric. Biotechnol. 30:101878. doi: 10.1016/j.bcab.2020.101878

[ref4] Dong M. Xiao Y. Yang B. Wang S. Sun L. Han Z.. (2024). *Serratia marcescens* AB1: a rhizosphere bacterium mitigating the acetochlor stress on the soil environment. Rhizosphere 30:100898. doi: 10.1016/j.rhisph.2024.100898

[ref5] El-Esawi M. A. Alaraidh I. A. Alsahli A. A. Alzahrani S. M. Ali H. M. Alayafi A. A.. (2018). *Serratia liquefaciens* KM4 improves salt stress tolerance in maize by regulating redox potential and stress-related gene expression. Int. J. Mol. Sci. 19:3310. doi: 10.3390/ijms19113310, PMID: 30355997 PMC6274875

[ref9002] Gu X. Zheng L. Zhai Q. Sun J. He H. Tang H.. (2022). Optimization of fermentation medium for biocontrol strain *Pantoea jilinensis* D25 and preparation of its microcapsules. Process Biochem. 121, 216–227. doi: 10.1016/j.procbio.2022.07.006

[ref7] Gupta P. He Y. Wang X. (2020). Interaction between herbicide residues and soil microbial communities: impacts on degradation and crop productivity. Front. Microbiol. 11:542. doi: 10.3389/fmicb.2020.00542, PMID: 32373080 PMC7186360

[ref8] Gupta A. Mishra R. Rai S. Bano A. Pathak N. Fujita M.. (2022). Plant growth-promoting bacteria mediated drought and salt stress tolerance in plants. Int. J. Mol. Sci. 23:3741. doi: 10.3390/ijms23073741, PMID: 35409104 PMC8998651

[ref9] Hamada M. A. Soliman E. R. S. (2023). Genomics identification of key genes involved in nitrogen cycling by *Serratia marcescens* OK482790. BMC Microbiol. 23:210. doi: 10.1186/s12866-023-02941-7, PMID: 37543572 PMC10403818

[ref10] Han Y. Ma H. Tao B. (2012). Stress Effects of diuron on germination and growth of maize seeds. Crop Sci. J. 2, 142–146. doi: 10.16035/j.issn.1001-7283.2012.02.008.2

[ref9003] He L. Wang Y. Liu R. Wang Y. Wang Y. Li Y.. (2025). Co-application of biochar and iron-based materials for remediating agricultural soil polluted with metolachlor and s-metolachlor: field research evidence. 2666–8211. doi: 10.1016/j.ceja.2025.100758

[ref12] Krueger M. C. Harms H. Schlosser D. (2015). Prospects for microbiological solutions to environmental pollution with plastics. Appl. Microbiol. Biotechnol. 99, 8857–8874. doi: 10.1007/s00253-015-6879-4, PMID: 26318446

[ref13] Kulkova I. Wróbel B. Dobrzyński J. (2024). Serratia spp. as plant growth-promoting bacteria alleviating salinity, drought, and nutrient imbalance stresses. Front. Microbiol. 15:1342331. doi: 10.3389/fmicb.2024.1342331, PMID: 38562478 PMC10982427

[ref14] Li Y. Chen Q. Wang C.-H. Cai S. He J. Huang X.. (2013). Degradation of acetochlor by consortium of two bacterial strains and cloning of a novel amidase gene involved in acetochlor-degrading pathway. Bioresour. Technol. 148, 628–631. doi: 10.1016/j.biortech.2013.09.038, PMID: 24075675

[ref15] Li H. Wang Y. Fu J. Hu S. Qu J. (2020). Degradation of acetochlor and beneficial effect of phosphate-solubilizing Bacillus sp. ACD-9 on maize seedlings. Biotech 10:67. doi: 10.1007/s13205-020-2056-2, PMID: 32030336 PMC6981330

[ref9004] Li J. Wen Y. Fang Z. Yang W. Song X. (2024). Application of cold-adapted microbial agents in soil contaminate remediation: biodegradation mechanisms, case studies, and safety assessments. RSC Adv. 14, 12720–12734. doi: 10.1039/d4ra01510j 38645519 PMC11027001

[ref9005] Liu Q. Jing W. Yang W. Huang M. Lu P. Hu D. (2024). Green manure (*Raphanus stativus* L.) alters soil microbial structure and promotes acetochlor degradation. Arab. J. Chem. 17:106017. doi: 10.1016/j.arabjc.2024.106017

[ref17] Liu H. Wang X. Ou Y. Cheng L. Hou X. Yan L.. (2023). Characterization of acetochlor degradation and role of microbial communities in biofilters with varied substrate types. Chem. Eng. J. 467:143417. doi: 10.1016/j.cej.2023.143417

[ref9006] Liu Y. Zhai Q. Lv J. Wu Y. Liu X. Zhang H.. (2025). Construction of a fusant bacterial strain simultaneously degrading atrazine and acetochlor and its application in soil bioremediation. Sci. Total Environ. 0048–9697. doi: 10.1016/j.scitotenv.2025.178478 39818196

[ref9007] Ma Q. Zhou Y. Parales R. E. Jiao S. Ruan Z. Li L. (2025). Effects of herbicide mixtures on the diversity and composition of microbial community and nitrogen cycling function on agricultural soil: A field experiment in Northeast China. Environ. Pollut. 372:125965. doi: 10.1016/j.envpol.2025.125965 40043878

[ref18] Mahdi I. Hafidi M. Allaoui A. Biskri L. (2021). Halotolerant endophytic bacterium *Serratia rubidaea* ED1 enhances phosphate solubilization and promotes seed germination. Agriculture 11:224. doi: 10.3390/agriculture11030224

[ref19] Novello G. Gamalero E. Massa N. Cesaro P. Lingua G. Todeschini V.. (2022). Proteome analysis of halotolerant *Serratia plymuthica* under salt stress. Microorganisms 10:890. doi: 10.3390/microorganisms10050890, PMID: 35630335 PMC9143289

[ref9008] Shakeel M. Hafeez F. Y. Malik I. R. Farid A. Ullah H. Ahmed I.. (2023). Serratia marcescens strain FA-4 enhances zinc content in rice grains by activating the zinc translocating enzymes. SABRAO J. Breed. Genet. 55, 495–507. doi: 10.54910/sabrao2023.55.2.21

[ref9009] Wang W. Shi H. Liu X. Mao L. Zhang L. Zhu L.. (2024). Enhanced remediation of acetochlor-contaminated soils using phosphate-modified biochar: Impacts on environmental fate, microbial communities, and plant health. Sci. Total Environ. 956:177359. doi: 10.1016/j.scitotenv.2024.177359 39500462

[ref9010] Xiao Y. Zheng L. Wang S. Dong M. Gao A. Han Z.. (2023). Bioremediation of metribuzin-contaminated soil by corn straw biochar-immobilized *Bacillus cereus* N1. Process Biochem. 520–533. doi: 10.1016/j.procbio.2023.05.006

[ref23] Xiao H. Zhou Q. Ma L. Q. (2005). Joint effects of acetochlor and urea on germinating characteristics of crop seeds. Sci. China Ser. C Life Sci. 48, 1–6. doi: 10.1007/BF02889795, PMID: 16089323

[ref24] Zhang M. Yu Z. Zhang M. Li X. Wang M. Li L.. (2022). *Serratia marcescens* PLR enhances lateral root formation through supplying PLR-derived auxin and enhancing auxin biosynthesis in Arabidopsis. J. Exp. Bot. 73, 3711–3725. doi: 10.1093/jxb/erac074, PMID: 35196372

[ref25] Zheng J. Liu C. Liu J. Zhuang J. Y. (2022). Bacterial-mediated legume growth promotion by *Serratia marcescens* X-45. Front. Microbiol. 13:988692. doi: 10.3389/fmicb.2022.988692, PMID: 36353452 PMC9638080

